# Inunction of Sapo Viridis in Scrofula

**Published:** 1882-02

**Authors:** 


### Inunction of Sapo Viridis in Scrofula.

Dr. Kormann, of Dresden, reports several cases of scrofula treated by the inunction of potash soap. The pathology of scrofula being essentially an overproduction and accumulation of lymphatic material in the lymphatic glands, treatment has aimed at the dispersion of this over-accumulation of lymph, before the process of cheesy metamorphosis sets in, a change to which such accumulations are especially prone, and which puts an end to the possibility of their removal by anything short of mechanical evacuation. The methods of treatment hitherto in use have not been eminently successful, and the opportunity for some advance in this direction is offered by the inunction of sapo viriditis. The author considers the good effect of such inunctions to be due possibly to the absorption of potash soap through the skin, and consequent liquefaction of the masses of lymphatic material by reason of direct contact with the soap. The inunctions were made once daily, just before bedtime, the amount of soap used being about a teaspoonful. The next morning the portion of the body to which the soap had been applied was carefully cleansed. In from three to four days, sometimes not until later, there was disquamation. A severe erythema was the exception, and eczema did not once result from the inunctions. As soon as much redness or itching was produced, the region chosen for inunction was changed, the skin nearest to the diseased glands or other lesion being used. Finally the soap was applied to the skin of the breast and back. In the meantime the skin directly over the diseased glands, to which the first applications of soap were made, had returned to its normal condition, and could be again used for inunction without causing pain. Under this treatment the infiltration of scrofulous glands disappeared with hitherto unknown rapidity, and the duration of scrofulous eczema also was decidedly shortened.—*Jahrbuch f. Kinderheilkunde.*

